# Shaking table test on seismic performance of a large-span high-rise building

**DOI:** 10.1038/s41598-024-57068-0

**Published:** 2024-03-19

**Authors:** Laite Sun, Yu Bai, Zhengcong Lai

**Affiliations:** 1 Faculty of Civil Engineering and Mechanics, Kunming University of Science and Technology, Kunming, Yunnan China; 2 Earthquake Engineering Researching Center of Yunnan, Kunming, Yunnan China

**Keywords:** Large-span high-rise building, Wave passage effect, Slope amplification effect, Scale model design, Shaking table test, Seismic response, Civil engineering, Engineering

## Abstract

This paper describes investigations in respect of the seismic performance of a large-span high-rise building in a mountainous area. The building consists of a 135 m high shear wall structure and a 174.5 m long steel truss structure, with dampers used to enhance the seismic performance. A 1/40 scale model of the prototype structure was designed, and shaking table tests was conducted. The experiments simulated the wave passage effect and slope amplification effect based on the building site and structural characteristics of the prototype structure. The seismic performance of the prototype structure was analyzed through the damage phenomenon, dynamic characteristics, and dynamic response of the model under earthquake effects. The results show that three seismic waves were delayed by about 0.4 s and amplified by about 1.6 times after passing through the steel frame with viscous dampers, which could effectively simulate the wave passage effect and slope amplification effect in the test. The maximum story drift ratios of the model shear wall structure and steel truss structure were 1/1258 and 1/455 for the SLE and 1/568 and 1/185 for the MCE. The damping devices played a key role in energy dissipation. As a result, this research provides a reference for the seismic design and shaking table testing of large-span high-rise buildings.

## Introduction

In recent decades, new types of super high-rise buildings have emerged one after another and become landmark buildings in many cities. Examples include the 101 Building (Taipei), the Shanghai World Financial Center (Shanghai), and the Hengda Building (Ningbo)^1–3^. In order to meet specific usage functions and achieve aesthetic styles, many super high-rise buildings combine the characteristics of large-span buildings, such as the CCTV Headquarters Building (Beijing), the Marina Bay Sands Hotel (Singapore), and the Island Tower Sky Club (Fukuoka)^4–6^. Compared with ordinary high-rise buildings, architects must consider the influence of seismic spatial effects, such as wave passage effects^7^, in these complex structures when designing lateral force-resistant systems, making it more challenging to analyze seismic performance.

The seismic performance analysis of existing structures has always attracted research interest in structural engineering, and researchers all over the world have employed various analyzing approaches for this subject^8–10^. The shaking table test is a common method for studying the seismic performance of complex high-rise buildings, it can load real earthquake acceleration records onto a scaled model of the prototype building to reproduce the seismic process. This is the most straightforward way to study the failure mechanism and various seismic response of complex high-rise buildings.

Lu et al.^11^ investigated the seismic performance of reinforced concrete frame-core tube structures, a common form of high-rise buildings. A reduced scale model (1/15 scale) of a typical reinforced concrete frame-core tube with a corner column removed from the first floor was designed, fabricated, and tested, and experimental results were presented regarding the seismic responses and actual process of collapse. Chen et al.^12^ designed and manufactured a 1/30 scale model of a long-span cantilevered story building, and then tested it via a shaking table facility to analyze the dynamic behavior, cracking pattern, and the likely governing failure mechanism of the structure. In order to study the seismic behavior of a 53-storey supertall building with a high-level transfer storey, Lu et al.^13^ conducted shaking table tests on a 1/30-scale model of a prototype structure. The model test results indicated that the prototype structure was able to withstand an earthquake of intensity 7 without severe damage. Guo et al.^14^ investigated an asymmetric high-rise building connected by two steel truss systems; the structural damage pattern and dynamic responses were analyzed via shaking table tests on a 1/45 scale model of the prototype structure. The results showed that the connecting trusses and rigid connection joints behaved well during strong seismic excitations, the structural damage was slight, and the structure presented high seismic resistance against strong ground motions. Lu et al.^15^ conducted shaking table tests on a 1/15 scale structural model of a high-rise building with two towers of different heights connected by trusses. The results showed that the whipping effect in the longitudinal direction developed sharply on top stories due to the stiffening action created by the connecting trusses, and structural responses at stories around the connecting trusses vary remarkably due to sudden changes in lateral stiffness.

**Figure 1:**
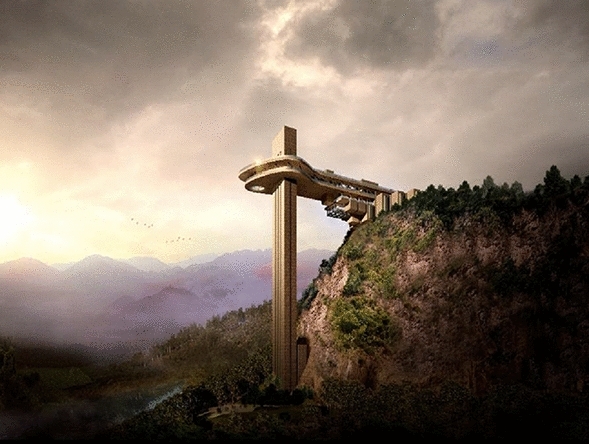
A large-span high-rise building (the cliff hotel).

The research object selected in this paper was a cliff hotel built in a mountainous area, a large-span high-rise building comprising a combination of a steel truss structure and a shear wall structure (Fig. 1). The large height and span of the building result in an undeniable time delay when seismic waves reach the supports at different heights along the mountain, which triggered the wave passage effect. The wave passage effect can affect the seismic response of buildings by generating different acceleration peaks and phases at different positions^16,17^. The current research results on the wave passage effect are mainly focused on common large-span structures such as large-span bridges, large-span space structures, dams and underground pipelines^18–21^, and there have been few theoretical studies or shaking table tests on the influence of the wave passage effect on large-span high-rise buildings. By calculating the floor displacements and the top floor maximum displacements of high-rise buildings, Li et al.^22^ showed that neglecting the wave passage effect will not provide an accurate structural response. To solve this problem, the traditional structural dynamics equations were modified using a time delay to incorporate the wave passage effect into the analysis of the structural response of high-rise buildings under seismic excitation.

Moreover, some structural supports of the cliff hotel investigated in this study are located at the top of the cliff, and the amplification effect of slope amplification occurs when seismic waves are transmitted longitudinally along the cliff to the top support^23^. In addition, the slope amplification effect can not be ignored in analyzing the seismic performance of buildings. Shabani et al.^24^ utilized 3D numerical analyses to investigate the seismic responses of three mid-rise buildings with 5, 10, and 15 stories in the vicinity of the slope crest, and the results indicated that the seismic responses of the 10- and 15-story buildings, including lateral displacements and base shear forces, were enhanced by the slope topography. Farghaly^25^ evaluated the seismic performance of city buildings constructed on rocky hillside slopes by studying base shear, acceleration, and displacements, and the results showed that these buildings would be severely damaged by earthquakes even if the peak ground acceleration magnitude was less than 0.25 g.

At present, the shaking table array is commonly used to simulate the wave passage effect, and the time difference is set for the seismic wave input to each shaking table in the array^26–28^. However, there is still a research gap regarding how to simulate the wave passage effect on a single shaking table. For the slope amplification effect, the relevant shaking table tests now mainly focus on the effect itself^29,30^, and there are no research results in respect of simulating the slope amplification effect via shaking table tests to study the seismic response of buildings. In order to access the seismic performance of the cliff hotel accurately using a shaking table test, it is necessary to simulate the wave passage effect and the slope amplification effect on a single shaking table. Therefore, in this study, a designed steel structure with viscous dampers was used to delay and amplify the input seismic waves in the test to simulate these effects.

In order to investigate the seismic performance of the prototype structure, a scale model (ratio 1:40) was subjected to shaking table test. The test simulated the time-delay and amplification effects of mountains on seismic waves based on the wave passage effect and slope amplification effect on the prototype structure. In this study, the seismic response of the model (including dynamic characteristics, acceleration response, and displacement), as well as the working conditions of the dampers, under the service level earthquake (SLE), design-based earthquake (DBE), and maximum considered earthquake (MCE) were tested by inputting white noise excitation and simulating seismic waves, to analyze and evaluate the seismic performance of the structure. In this study, a shaking table test of a high-rise building, which considers the wave passage effect and slope amplification effect, fills the gap in the research field regarding the seismic performance of high-rise buildings, particularly large-span high-rise buildings, as well as simulating the wave passage effect and slope amplification effect using a single shaking table. The test conclusions can be applied to the structural design and seismic design of similar high-rise buildings in mountainous areas, and can help to improve the sustainability of building safety. The method used in the test to delay and amplify the input seismic waves can be applied in future shaking table tests that need to simulate wave passage effects and slope amplification effects.

## The prototype structure

**Figure 2:**
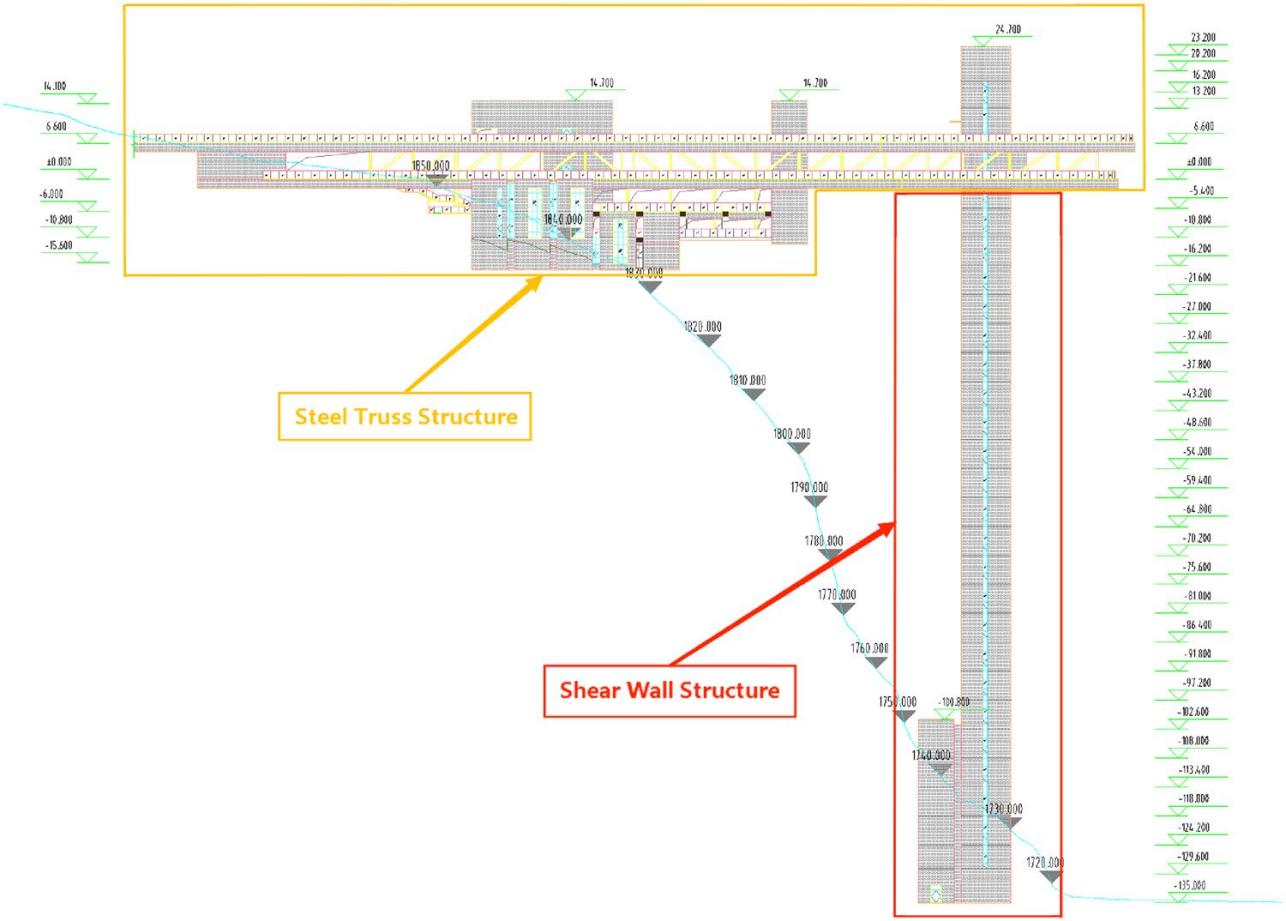
The structural system.

The prototype structure is built in the mountainous area of Yunnan Province, China, and is about 155.3 m high. It consists of a combination of a high-rise shear wall structure and a large-span steel truss structure (Fig. 2). The shear wall structure is 135 m above the ground with 25 floors, and the steel truss structure is 174.5 m long with a span of 70.5 m. One end of the steel truss structure is connected to the top of the shear wall structure, and the other is on the mountain. The vertical force of the steel truss structure is directly transmitted to the mountain at one end and to the shear wall structure at the other. According to current Chinese Code GB 18306-2015^31^, the seismic acceleration peak of the proposed site is 0.3 g. The characteristic site period is 0.45 s, and the seismic intensity is 8 degree.

### Shear wall structure

**Figure 3:**
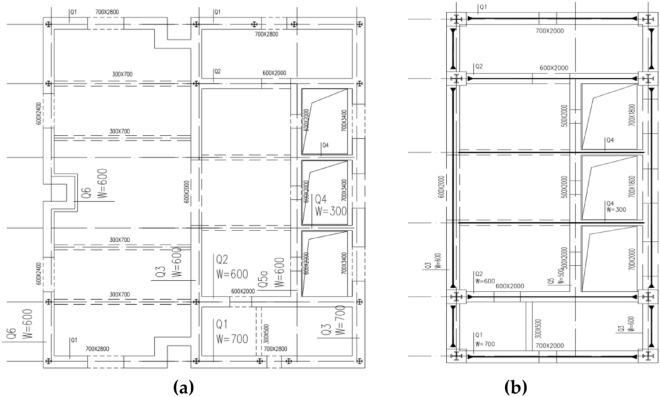
Plan layout of shear wall structure. (**a**) Base-6 floors; (**b**) floors 7–25.

The shear wall structure serves as the main vertical and lateral resisting components. The structural plane is rectangular-shaped, with the size of 20.4 m × 17.6 m (Base-6 floors) and 20.4 m × 9.6 m (floors 7–25), and the floor height is 5.4 m (Fig. 3). The shear wall structure bears both vertical and horizontal loads, and concrete provides excellent structural stiffness, exhibiting good torsional performance under horizontal loads. To ensure that the reaction force can be transmitted to the shear wall structure, steel structure concealed columns and steel concealed beams are installed on the transition floors between the 24th and 25th floors. The thickness of each floor slab is 120 mm, using cast-in-place reinforced concrete floor slabs with double-layer bidirectional reinforcement. Due to the rigid connection between the top and the steel truss with a bending moment at the top and a reverse bending point in the middle, the entire wall is of the same thickness.

### Steel truss structure

**Figure 4:**
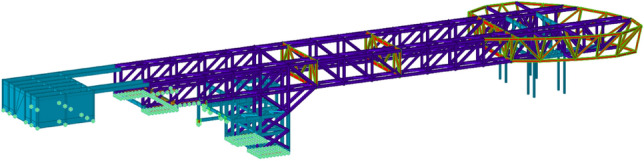
3D graph of steel truss structure.

The main part of the steel truss structure is combined by two tetrahedral steel trusses with a 6.6 m height (Fig. 4). Due to the reliability and integrity of the foundation-connected mountain and the principle of direct force transmission, the main horizontal force is borne by the rear support. In addition, the number of front supports is minimized to reduce horizontal force transmission. Due to the temperature caused by the large internal force, the main truss is made of high-strength steel, which can not only meet the bearing capacity requirements but also reduce temperature stress. The floor structure comprising the main beam of the floor is arranged perpendicular to the direction of the steel truss, and the secondary beam is arranged parallel to the direction of the steel truss. The secondary beam is designed as a composite beam.

### Seismic design

As a tourism facility, the prototype structure is a large public building with dense personnel. It is located in a high-intensity seismic fortification area with frequent earthquakes. According to the provisions of the current Chinese Code GB50011-2010^32^, shock absorption technology must be adopted to enhance earthquake resistance capacity of the building. According to GB50011-2010 and JGJ3-2010^33^, the target of this structural seismic design is as follows: The structure must remain fully elastic without the destruction of the structural components, which must not be damaged under the action of an SLE. Under the action of a DBE, the structure may be damaged and can still be used after general repairs, resulting in minor damage to the structural components. Under the action of an MCE, the structure does not collapse or undergoes severe damage, and the structural components undergo mild to moderate damage (Table 1).

**Table 1:** The target of seismic performance.

Seismic intensity level	SLE	DBE	MCE
Qualitative description of performance levels	No damage	Minor damage	Moderate damage
*The maximum story drift ratios*			
Steel truss	1/278	–	1/100
Shear wall	1/1112	–	1/200

**Figure 5:**
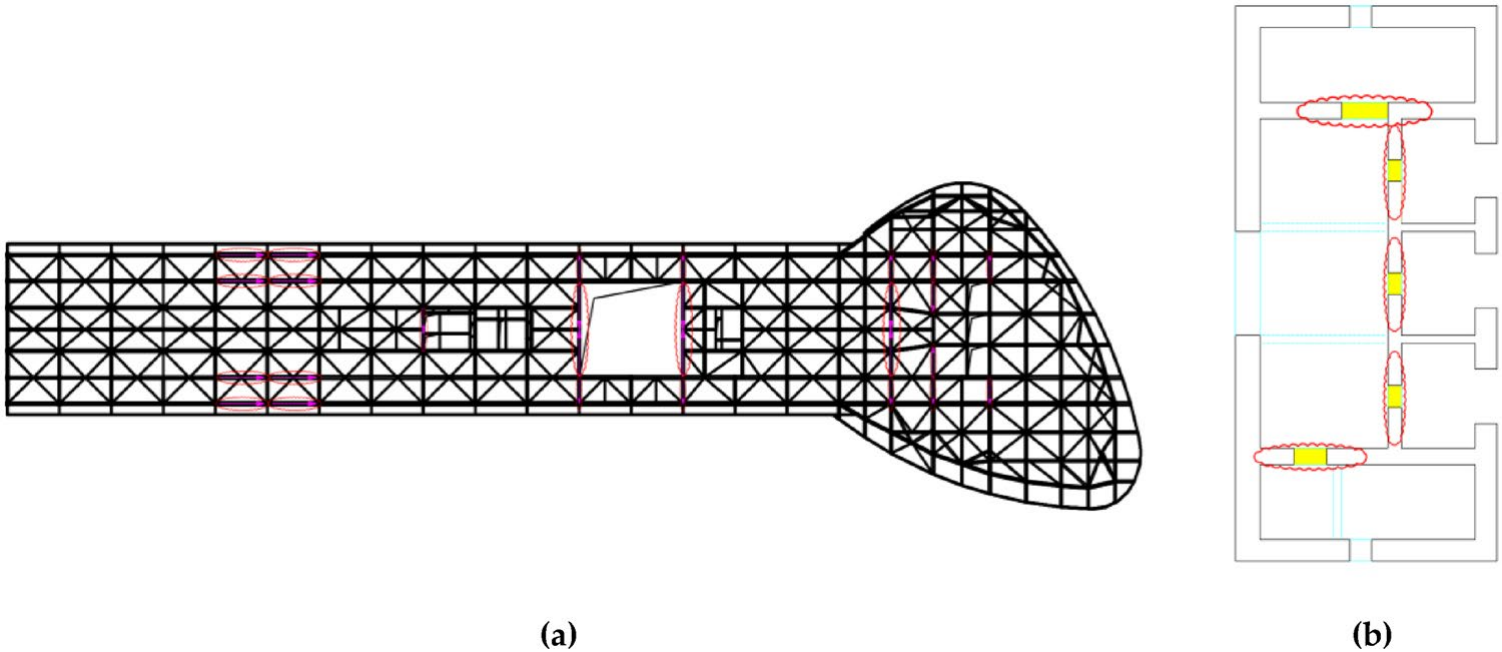
Layout of damping devices. (**a**) BRBs; (**b**) CBDs.

In this study, based on the function of the structure, site conditions, the importance of components, and economic factors, buckling restrained braces (BRBs) and coupling beam dampers (CBDs) were ultimately selected as the seismic design scheme for damping devices. Under the action of an SLE, the BRBs and CBDs did not yield and provided stiffness for the structure. Under the action of a DBE, the BRBs and CBDs partially yielded and dissipated energy, while the BRBs and CBDs all yielded and dissipated energy under the action of an MCE. In detail, BRBs were installed at the connection layer of two different structures to strengthen the integrity of the overall structure, and were used to further strengthen against the horizontal stress around the opening of this floor (Fig. 5a). BRBs were also installed in the other layers of the steel truss structure where the deformation was large. CBDs were added at the significant deformation position in the lower part of the shear wall structure (Fig. 5b). Where ρ is the mass density of materials, and A is the equivalent unit cross-sectional area.

### Wave passage effect and slope amplification effect

The prototype structure was built at the mountain area. The height difference from the bottom of the mountain to the top where the steel truss structure was supported was about 141 m, with a terrain slope of 40 degree to 50 degree. According to GB 50011-2010, the slope amplification effect must be considered when calculating the seismic response of structures. Based on the height difference and slope of the mountain, the amplification coefficient of seismic wave acceleration was determined to be 1.6 according to GB 50011-2010 and relevant literature^34^. In other words, the peak acceleration of the input seismic wave at the bottom of the slope was amplified by 1.6 times when it reached the top.

**Figure 6:**
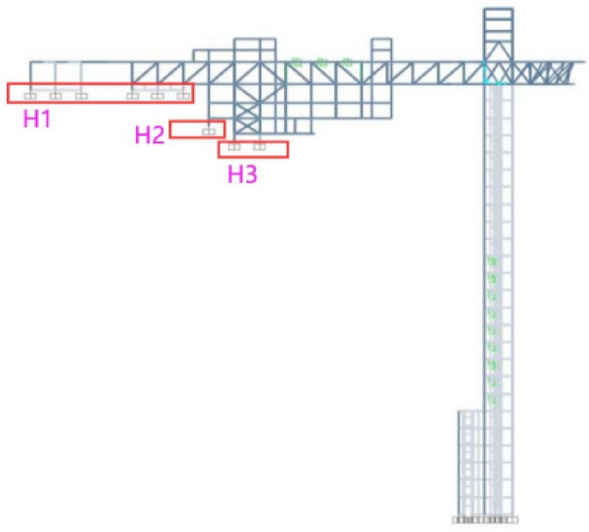
Structural supports.

For large-span structures, it is necessary to regard the impact of wave passage effects on the prototype structure. Since the wave passage effect is caused by the time-delay of seismic waves reaching different supports of the building, researchers set the time difference of seismic waves input at different supports to reflect the wave passage effect in calculating the seismic response. Based on the geological survey data, the equivalent shear wave velocity of the mountain was calculated as V_se_ = 339 m/s based on the weighted average value. The slope top supports were divided into three groups according to the elevation, and the height difference between the slope top supports and the bottom supports was H_1_ = 133 m, H_2_ = 122 m, H_3_ = 117 m (Fig. 6). The time t for seismic waves to propagate along the mountain from the bottom to the top of the slope was equal to the height difference H divided by the equivalent shear wave velocity V_se_. Since the time difference calculation results for the three groups of supports were approximately equal to 0.4 s (t_1_ = 0.39 s, t_2_ = 0.36 s, t_3_ = 0.35 s), the final time difference was uniformly taken as 0.4 s.The supports at the top of the slope were delayed by 0.4 s in respect of inputting seismic waves compared to the supports of the shear wall structure.

## Shaking table test

The experiment was conducted at Yunnan Seismic Engineering Technology Research Center in China, using a seismic simulation shaking table device. The main parameters of the shaking table device are shown in Table 2.

**Table 2:** Main parameters of the shaking table device.

Item	Value	Item	Value
Size	4.0 m × 4.0 m	Maximum acceleration	± 1 g (payload: 20t)
Maximum payload	30t		± 0.8 g (payload: 30t)
Maximum displacement	± 125 mm	Maximum velocity	± 0.8 m/s
Working frequency	0.1–50 Hz		

### Model design

It was the most crucial task to determine the similarity relationship between model structure and prototype structure in the shaking table test. Since many physical quantities in the shaking table test research in respect of high-rise building structures simulating earthquakes make it impossible to determine a clear functional relationship between the physical quantities, the dimensional analysis method is widely used. Firstly the dimensional analysis method determines the similarity conditions, and then derives the remaining similarity scaling factors from the controllable similarity scaling factors to complete the similarity design. Based on the principle of dimensional coordination^35^, the relationship of similarity scaling factors such as the elastic modulus, density, length, and acceleration is as followed:

1 $$ S_{E} /S_{\rho } S_{a} S_{L} = 1 $$

where S_E_, S_ρ_, S_a_ and S_L_ are similarity scaling factors for the elastic modulus, density, acceleration, and length.

The length similarity scaling factor, S_L_, is usually established first. Large shaking table test models are convenient for construction, and the impact of size effects is relatively small. Hence, the model should be as large as possible. In other words, S_L_ should be taken to be as large as possible. However, researchers should ensure the geometric dimensions of the model plane are within the range of the shaking table surface. In addition, the elevation of the facade should meet the height requirements of the laboratory production site and the model lifting crane.

In this study, considering the dimensions of the laboratory and the shaking table device, S_L_ was taken as 1/40. The elastic modulus similarity scaling factor S_E_ was determined by the characteristics of the model material used. According to the laboratory conditions and similarity requirements, we used micro-concrete to simulate the concrete of prototype building, galvanized iron wire for the steel reinforcement, and red copper for the steel components. These material mechanical properties are shown in Table 3. According to material performance testing, S_E_ was taken as 0.2. The acceleration similarity scaling factor S_a_ was determined by factors such as the maximum ground acceleration peak, shaking table noise, and table bearing capacity, ranging from 1 to 3. According to the various parameters of this experiment, S_a_ was taken as 1.8. The density similarity scaling factor S_ρ_ was determined using the first three similarity scaling factors through formula (3.1). However, the obtained density similarity relationship often required the model material to have a high density. In addition, based on the stress similarity constant mentioned above, the material was required to have a lower elastic modulus, which was difficult to achieve. Therefore, the equivalent density of the model was increased by configuring counterweight blocks in model production.

**Table 3:** Mechanical properties of model materials.

Material	Yield strength/MPa	Ultimate strength/MPa	Elastic modulus/MPa
Micro-concrete	13.18	–	7952
Galvanized iron wire	280	375	2.06 × 10^5^
Red copper	70	200	1.07 × 10^5^

The main similarity scaling factors of this test were calculated using dimensional analysis, shown in Table 4.

**Table 4:** Main similarity scaling factors.

Parameter	Relation	Scaling factors
Length	S_L_	0.025
Elastic modulus	S_E_	0.2
Acceleration	S_a_	1.8
Density	S_ρ_ = S_E_/(S_a_S_L_)	4.44
Stress	S_σ_ = S_E_	0.2
Mass	S_m_ = S_ρ_S_L_3	0.0000694
Concentrated force	S_F_ = S_σ_S_L_2	0.000125
Frequency	S_f_ = (S_a_/S_L_)0.5	8.485281
Strain	S_ε_	1
Period	S_T_	0.117851

**Figure 7:**
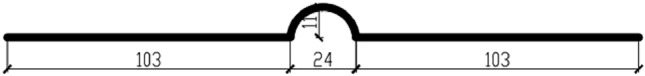
BRB1 model size.

The BRB model was designed based on the principle of similarity in yield force and displacement. Taking the BRB1 model as an example, a red copper rod was selected as the simulation component and made into the form shown in Fig. 7. The yield of the BRB was simulated using the yield at the vertex of the bending part in the middle of the component. In addition, the yield force and displacement of the simulated component were adjusted to meet the design requirements of a similar yield force and displacement by adjusting the curvature of the curved part. The performance parameters of the BRB model are shown in Table 5. Due to the small size of the CBD after scaling according to the similarity scaling factor, it could not be processed and manufactured. Therefore, a coupling beam made of particle concrete and galvanized iron wire was used for equal substitution in the model.

**Table 5:** BRB model performance parameters.

Type	Yield force/N	Yield displacement/mm	Quantity
BRB1	225	0.2	4
BRB2	225	0.18	1
BRB3	225	0.163	3
BRB4	250	0.143	4
BRB5	375	0.193	8
BRB6	225	0.1	4
BRB7	450	0.178	2
BRB8	625	0.213	6
BRB9	500	0.16	12
BRB10	500	0.155	10
BRB11	500	0.143	2
BRB12	750	0.203	16
BRB13	563	0.135	4
BRB14	875	0.12	8
BRB15	2250	0.178	4

### Seismic wave selection, time-delay and amplification

**Figure 8:**
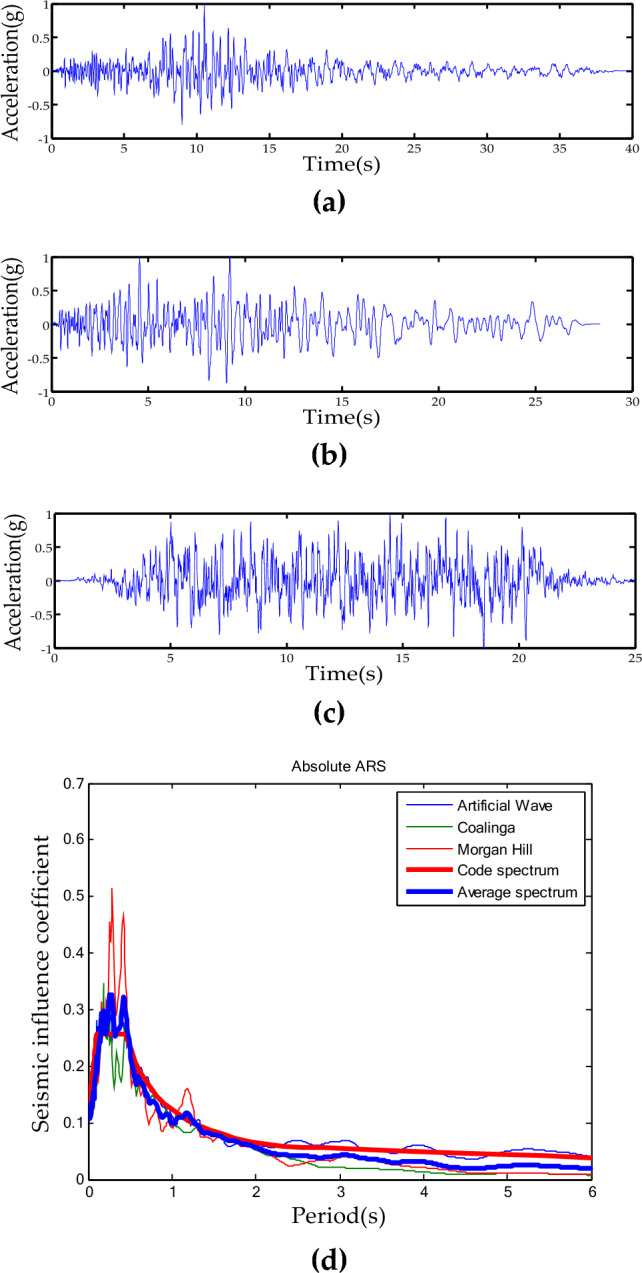
The input seismic waves. (**a**) The Coalinga wave; (**b**) the Morgan Hill wave; (**c**) the artificial wave; (**d**) response spectrum.

According to GB 50011-2010, two actual seismic waves (the Parkfield Vineyard record of the Coalinga earthquake and the San Justo Dam record of the Morgan Hill earthquake) and one artificially-simulated seismic wave were selected as the test input seismic wave. The time data and response spectrum of the three seismic waves are shown in Fig. 8.

The input seismic wave needed to be amplified and delayed to simulate the slope amplification effect and wave passage effect in this shaking table test. Based on the actual situation of prototype structure described above, a 0.4 s time-delay of the seismic waves should be provided in the span direction of steel truss structure during this shaking table test, and the peak acceleration of the seismic waves at the top of structure should be amplified by 1.6 times. Considering the large amount of seismic wave acceleration data and existing experimental techniques, it is not possible to amplify and delay all peak accelerations. Therefore, the test was further simplified to focus only on the delay and amplification of the maximum peak acceleration.

**Figure 9:**
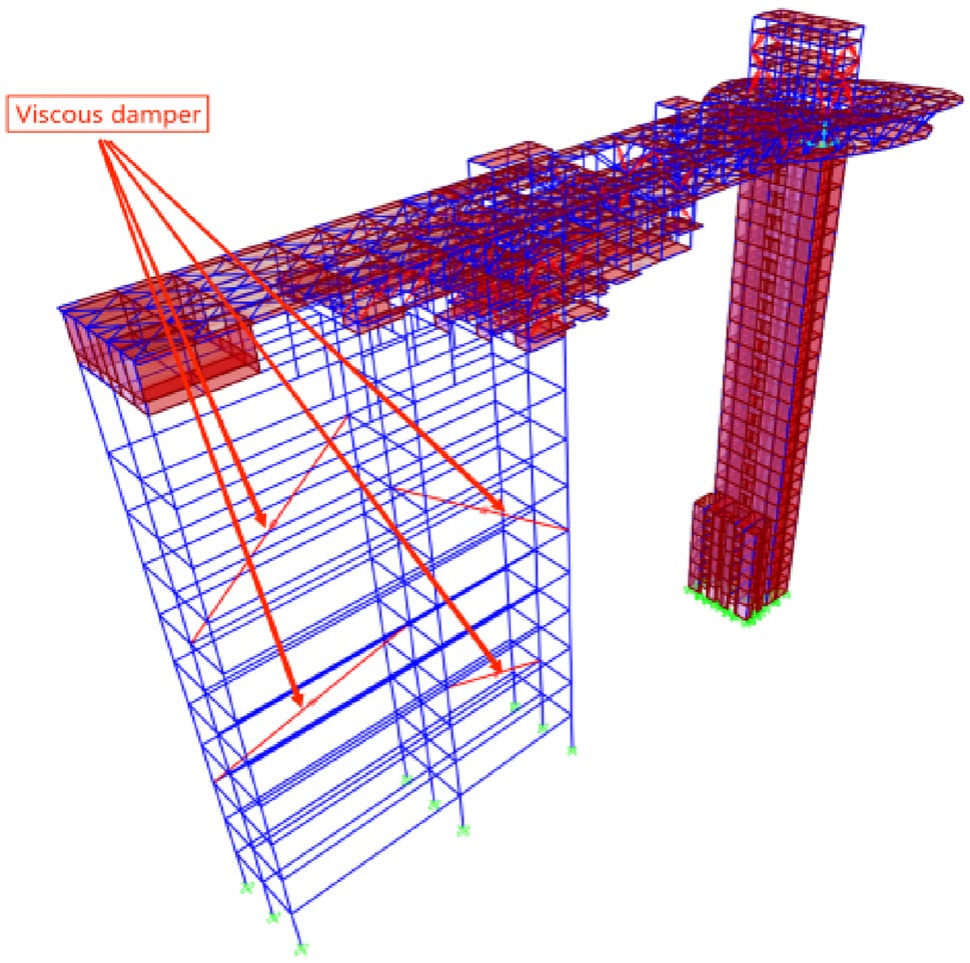
Steel frame structure and prototype structure.

The higher the height and stiffness of a structure, the more pronounced the amplification effect of seismic waves at its top. Based on practical engineering experience, installing viscous dampers could cause a significant time-delay effect on seismic waves. Installing more viscous dampers could cause a more apparent time-delay effect. In addition, increasing the number of dampers could decrease the amplification effect of seismic waves. For the convenience of subsequent shaking table tests, the use of a steel frame with viscous dampers was proposed to amplify and delay seismic waves based on various factors^36^. The size of the steel frame structural components and the number of viscous dampers were continuously adjusted to balance the time delay and amplification effects, achieving the expected design goals of steel frame structures. When designing steel frame structures, the overall finite element models of the prototype structure and steel frame structure were first established (frame element for beams and columns, thin shell element for slabs, nonlinear multi-layer shell element for shear walls). Concrete C30 was adopted for beams and slabs, while C60 for shear walls and columns. Normal rebar was employed with HPB335 and HRB400, and profile steel was employed with Q355. Then, the three selected seismic wave original acceleration records were inputted into the model for multiple time history analyses. Since there is no mature application law for the time delay effect of dampers on seismic waves, extensive finite element analysis was required to obtain the optimal configuration of dampers. On the premise that the steel frame structure could achieve the expected delay and amplification effects of seismic waves, the appropriate size of the steel frame structure components and the number, position and parameters of viscous dampers were determined (Fig. 9). The beam of steel frame was selected with a size of 2500 mm × 1500 mm × 400 mm × 400 mm box girder with steel frame column size of 3500 mm × 2500 mm × 500 mm × 500 mm box shaped column. Four sets of viscous dampers were installed between floors, with a damping coefficient of 400kN/(mm/s)^α^ (α = 0.25). The span direction of steel truss structure was defined as the X direction, and the perpendicular direction of X direction was defined as the Y direction. The results of seismic wave delay and amplification analysis are shown in Table 6.

**Table 6:** Time-delay of seismic wave and amplification of peak acceleration.

Seismic wave and direction	Time delay/s	Acceleration amplication
Artificial wave (X)	0.36	1.63
Artificial wave (Y)	0.06	1.68
Coalinga wave (X)	0.39	1.57
Coaling wave (Y)	0.08	1.55
Morgan Hill wave (X)	0.42	1.82
Morgan Hill wave (Y)	0.07	1.71

### Model construction

**Figure 10:**
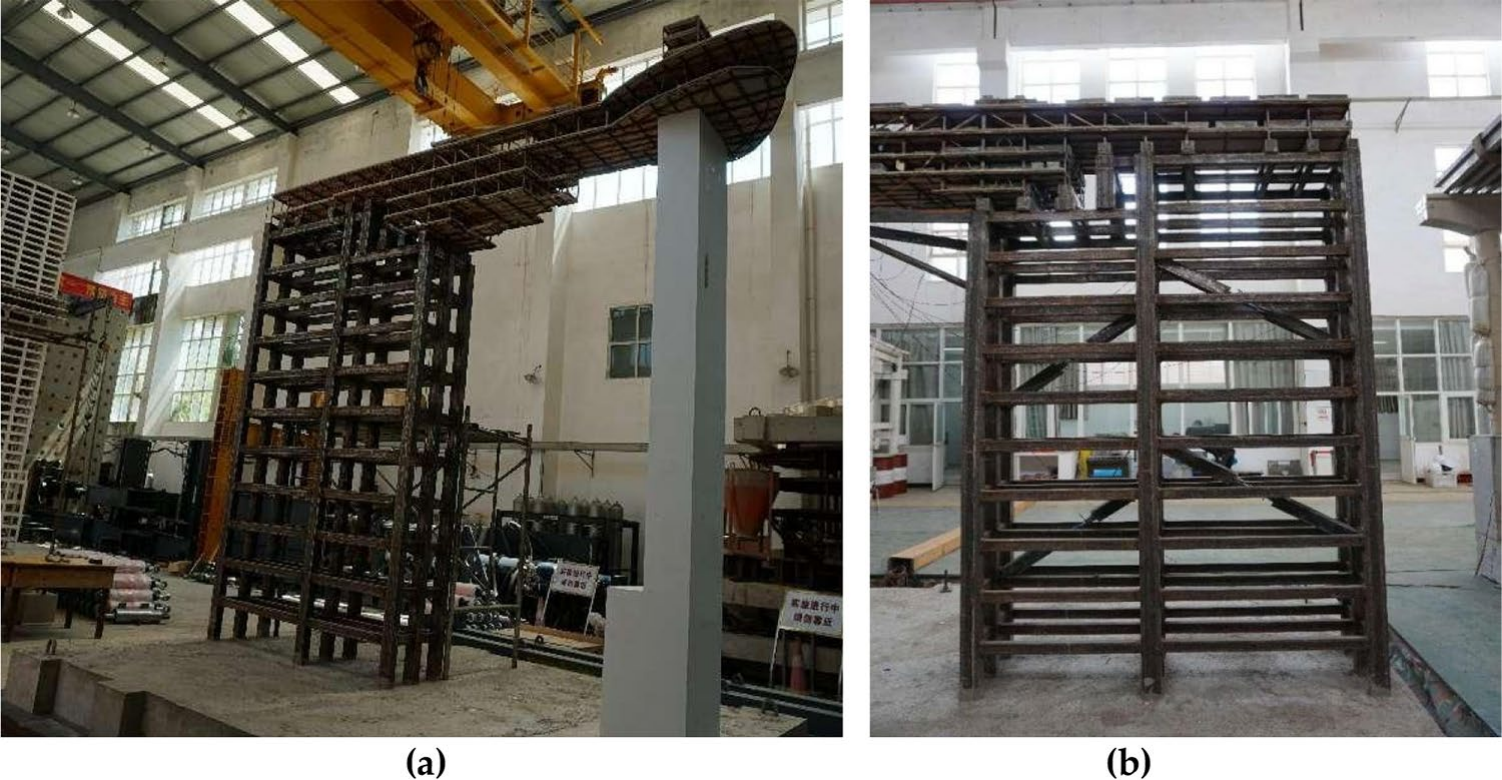
Complete scale model. (**a**) prototype model and steel frame; (**b**) steel frame with viscous dampers.

The components of prototype building were complex and numerous, meanwhile the reduction of model space brought difficulties to construction. Therefore, when constructing scale model, appropriate simplification needed to be achieved without changing the structural mechanism. The scale model eliminated non-main structural components, e.g. guardrails. Some load-bearing components were simplified, e.g., the consolidation of shear wall openings. The total height of the scale model was 3.88 m, with a total weight of 2.726t, including 0.65t of self-weight and 2.076t of counterweight (Fig. 10).

### Testing instruments and arrangements

**Figure 11:**
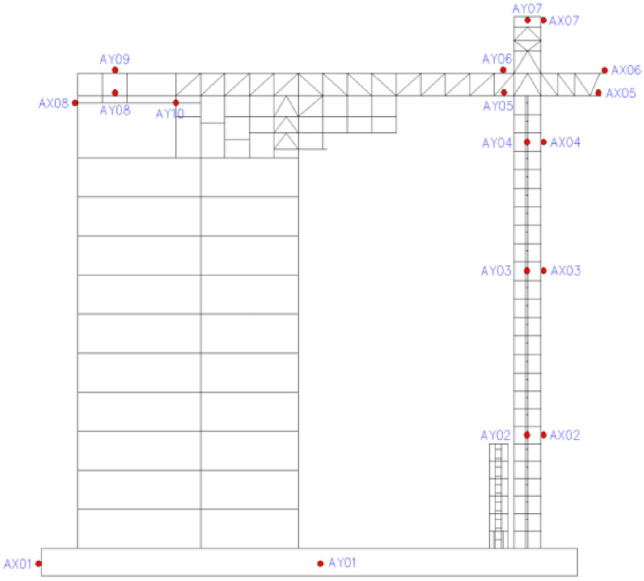
Layout of accelerometer.

In this test, researchers used a variety of test instruments to measure the experimental seismic response of the scale model, such as dynamic signal acquisition system, accelerometer, resistance strain gauge, and data processing computer. A total of 18 accelerometers were arranged in the experiment to record the acceleration response, which were installed in the X and Y directions of the base, the top of the steel frame, and the 7th, 16th, 23rd, 25th, 26th, and 30th floors (Fig. 11). The letter A refers to accelerometers, and the letters X and Y refer to the X direction and Y direction, respectively. In order to investigate the stress and strain values of components with complex stress under the earthquake load, resistance strain gauges were attached to shear walls, steel trusses, BRBs, and other locations for strain testing to monitor the stress and elastic–plastic deformation of essential parts.

### Loading history

The model structure was loaded under various seismic conditions in the order SLE (PGA = 0.20 g), DBE (PGA = 0.54 g), and MCE (PGA = 0.92 g) (Table 7). The loaded acceleration records were obtained by adjusting the peak values of the three selected seismic wave acceleration versus time datasets, based on the peak acceleration values and similarity relationships that the prototype structure should adopt under various seismic actions and compressing the excitation time. To investigate the dynamic characteristics and its change rules in different phases, before and after the input of the earthquake action of different intensities, a white noise frequency sweep was carried out to test the changes in structural dynamic characteristic parameters of the model.

**Table 7:** Loading cases.

Phase	Seismic wave	Input direction	Input PGA/g
White noise	–	X,Y	0.11,0.11
SLE	Coalinga wave	X	0.20
		Y	0.20
	Morgan Hill wave	X	0.20
		Y	0.20
	Artificial wave	X	0.20
		Y	0.20
White noise	–	X,Y	0.11,0.11
DBE	Coalinga wave	X	0.54
		Y	0.54
	Morgan Hill wave	X	0.54
		Y	0.54
	Artificial wave	X	0.54
		Y	0.54
White noise	–	X,Y	0.11,0.11
MCE	Morgan Hill wave	X	0.92
		Y	0.92
	Artificial wave	X	0.92
		Y	0.92
White noise	–	X,Y	0.11,0.11

## Experimental result and analysis

### Test phenomenon

**Figure 12:**
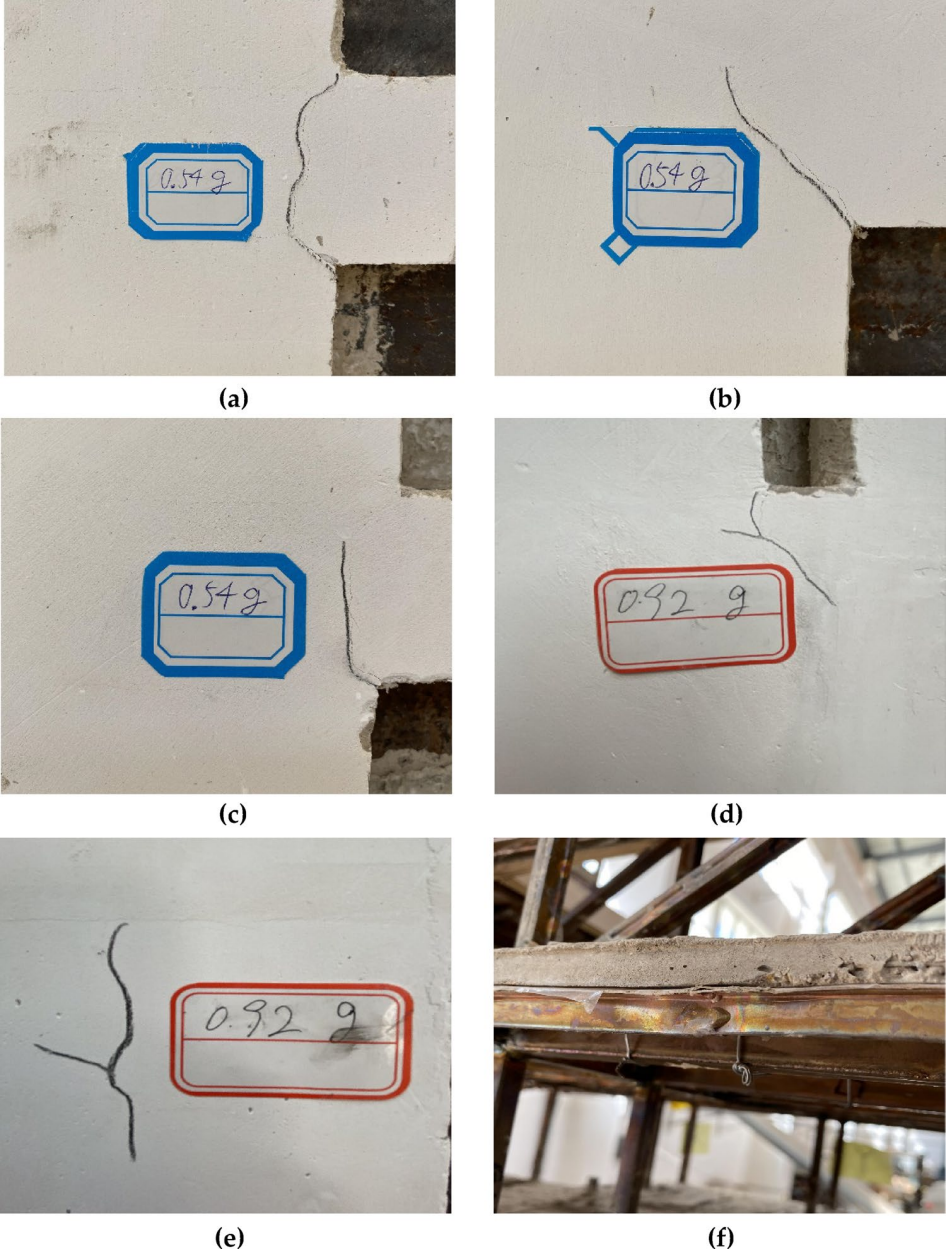
Model construction process: (**a**) crack at connecting beam of 6th floor; (**b**) crack at corner of the opening of 14th floor; (**c**) crack at corner of the opening of 19th floor; (**d**) crack at corner of the opening of 10th floor; (**e**) crack at shear wall of 17th floor; (**f**) local buckling at 26th floor.

The model underwent SLE, DBE, and MCE simulations in sequence, with the peak acceleration gradually increasing from 0.20 to 0.92 g. The phenomenon of structural testing is described as follows:

1. Under the SLE action, no obvious cracks were found on the components, indicating that the model stayed in the elastic condition, and the performance goals of seismic design were meeting.
2. Under the DBE action, many slight cracks appeared at the ends of some connecting beams, as well as the corners of the openings in the shear wall structure of the model, indicating some damage inside the model (Fig. 12a–c).
3. Under the MCE action, the displacement response was quite obvious, and cracks at the ends of the connecting beams and the corners of the openings deepened. The cracks gradually increased, and some shear walls exhibited horizontal and vertical cracks (Fig. 12d–f). Some components of the steel truss structure exhibited yield deformation, and the model had a reasonable yield mechanism. Some components of the model entered a plastic state, but the overall integrity of the model was good, and the shock absorption system could still function. The structure had considerable load-bearing capacity.

### Dynamic characteristics

The natural frequencies of the scale model in different test phases could be obtained by sweeping it with white noise before and after earthquakes of different intensities (Table 8). The results were analyzed and the results are as follows:

1. The 1st and 2nd order modes were translational, while the 3rd order mode was torsional. The 1st order frequency was clearly lower than 2nd order, which means the model stiffness was much stronger in the Y direction than in the X direction. The fthree frequencies of the model remained essentially constant after the SLE action, showing that the model was still in the elastic condition.
2. After the DBE action, the first three natural frequencies of the model continued to decrease, with a significant decrease in the first frequency, indicating that some components were in an inelastic state and the model underwent some damage.
3. After the MCE action, the first three natural frequencies of the model significantly decreased, with a decrease rate of more than 10%. The model entered the elastic–plastic state and the lateral stiffness of the model significantly degraded. The degradation rate of the model stiffness in the X direction was greater than in the Y direction, but the overall seismic bearing capacity remained good.

**Table 8:** Change of dynamic characteristics.

Phase	Order		
	1st	2nd	3rd
Initial			
Frequency/Hz	4.83	14.24	16.46
Reduction	–	–	–
After SLE			
Frequency/Hz	4.81	14.23	16.46
Reduction	0.6%	0.1%	–
After DBE			
Frequency/Hz	4.29	13.17	15.39
Reduction	11.28%	7.52%	6.48%
After MCE			
Frequency/Hz	3.94	12.49	14.62
Reduction	18.44%	12.28%	11.17%

### Time-delay and amplification

By comparing the acceleration data taken by the accelerometers put on the model base and the steel frame top, the time-delay and amplification data generated by the greatest peak acceleration of seismic waves when transmitted to the steel frame top were obtained (Table 9). The experimental results basically met the expected goals of seismic wave time-delay of 0.4 s in the X-direction, while amplification of the maximum peak acceleration at the steel frame top by 1.6 times. In summary, the test used a design steel frame to produce the desired time-delay and amplification effects of the input seismic waves.

**Table 9:** Time-delay of seismic wave and amplification of peak acceleration.

	Sensor location	Coalinga		Morgan Hill		Artificial wave	
		X	Y	X	Y	X	Y
Moment of maximum peak acceleration/s	Model base	5.83	5.60	5.68	6.27	7.28	5.75
	Steel frame top	6.27	5.71	6.03	6.36	7.61	5.81
Time-delay/s		0.44	0.11	0.35	0.09	0.33	0.06
Maximum peak acceleration/m·s^−2^	Model base	2.19	1.87	2.13	1.90	2.06	1.85
	Steel frame top	3.74	3.29	3.91	2.93	3.19	2.99
Acceleration amplication		1.71	1.76	1.84	1.54	1.55	1.62

### Acceleration response

**Figure 13:**
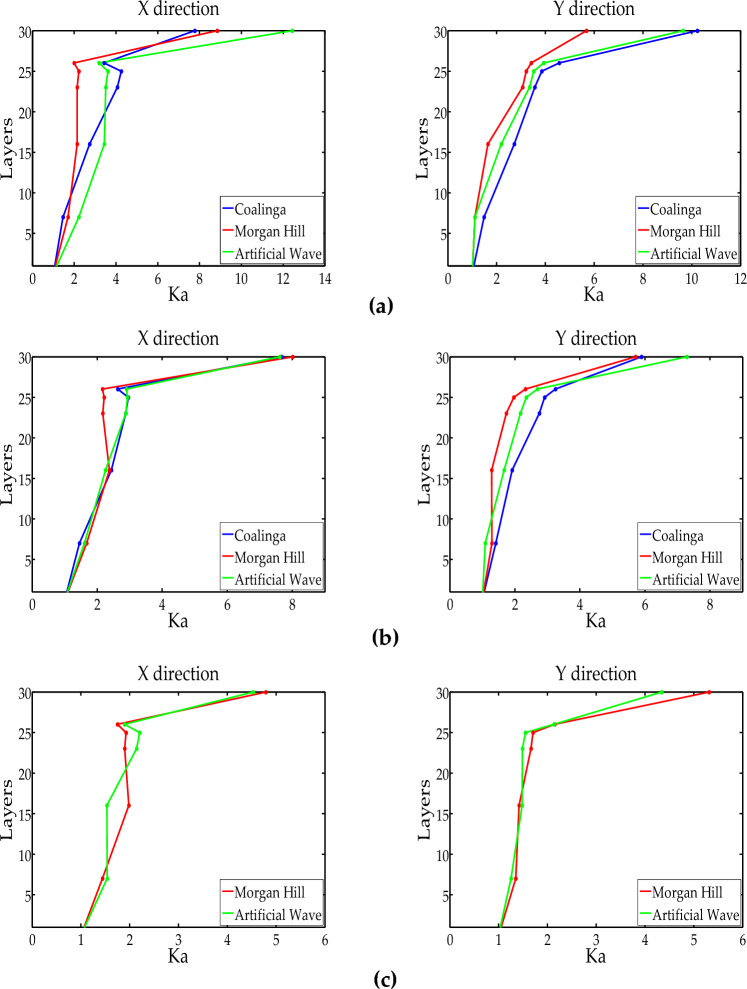
Model acceleration response: (**a**) SLE; (**b**) DBE; (**c**) MCE.

The acceleration response was represented by K_a_, K_a_ = PFA/PGA(PFA is peak floor acceleration). Based on the acceleration data, the variation of K_a_ under different levels of seismic action is shown in Fig. 13. From the analysis, the following can be seen:

1. The K_a_ ranged from 1 to 12.5, with the acceleration response in the Y direction greater than in the X direction, and the acceleration response at the top being the highest. Due to the steel truss structure causing sudden change in the vertical lateral stiffness, the whipping effect on the top occurred. The whipping effect refers to the phenomenon where the amplitude of the slender protruding part of the top of a high-rise building increases dramatically under seismic action.
2. Under different seismic wave excitation of equal intensity, the shapes of K_a_ curves were different due to the various spectral characteristics of the seismic waves, but the trend of curves was consistent. As the PGA increased under different seismic actions, the acceleration amplification coefficient of the structure gradually decreased. This phenomenon indicated that the damage to the structure gradually deepened, and the lateral stiffness gradually deteriorated.

### Displacement response

**Figure 14:**
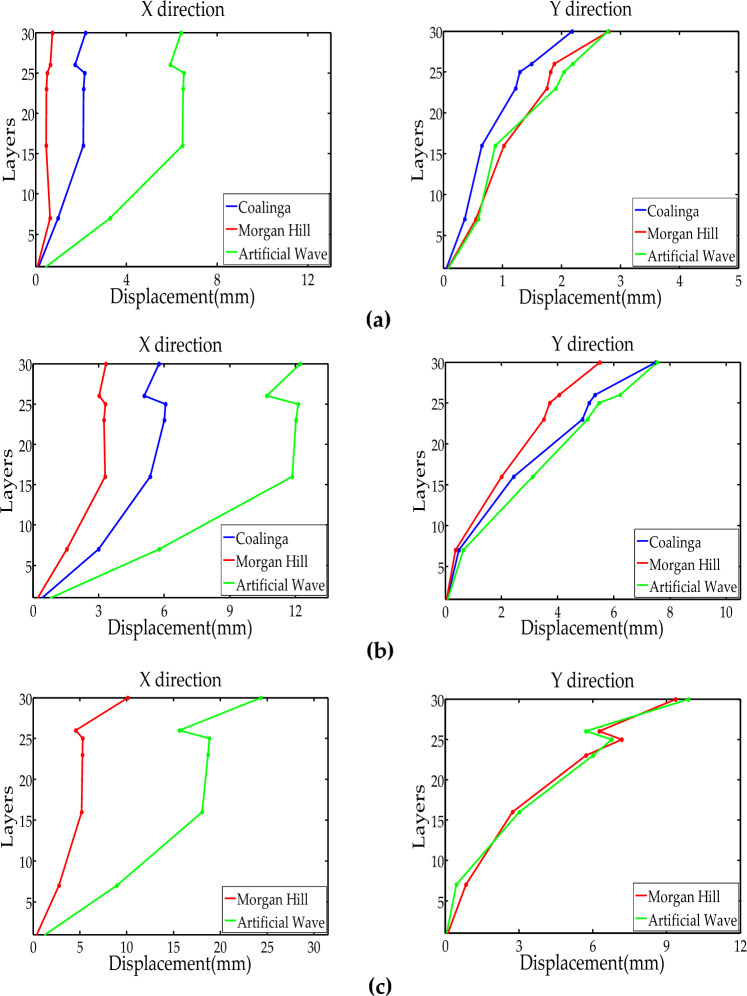
Model displacement response: (**a**) SLE; (**b**) DBE; (**c**) MCE.

The displacement data was obtained by quadratic integration of acceleration data. Before the test, a comparison was made between the integral displacement at the same measuring point and the results collected by the displacement sensor. Stable agreement between the two indicated that the integral displacement results were stable. Therefore, the displacement data results in the experiment were analyzed through integral displacement. The maximum lateral displacement curve relative to the base under different levels of seismic action is shown in Fig. 14. The specific rules are as follows:

1. With the enhancement of PGA, the lateral displacement increased gradually. The lateral displacement curve under the 26th floor was relatively smooth, indicating that the lateral stiffness of shear wall structure was distributed evenly. The 26th floor was a steel truss structure with BRBs, which lead to a marked change in lateral stiffness, resulting in a clear turning point in the curve.
2. Under different levels of earthquake action, the displacement response of the model structure in the Y direction was smaller than that in the X direction, indicating that the lateral stiffness of the structure in the Y direction was greater than that in the X direction. The lateral displacement of the model under the MCE action was significantly greater than that of the SLE and DBE, and the increasing rate was also faster than for the SLE and DBE. This finding indicates that the lateral stiffness significantly decreased under the MCE action, which was consistent with the degradation of structural frequency.

Under the SLE action, the maximum story drift ratios in the X and Y directions of the model shear wall structure were 1/1258 and 1/1341, which were less than the requirement of the SLE displacement angle limit of 1/1112. The maximum story drift ratios of the steel truss structure were 1/821 and 1/455 in the X and Y directions, which were less than the requirement of the SLE displacement angle limit of 1/278. This finding demonstrates that the structure met the seismic performance target under the SLE. Under the MCE action, the maximum story drift ratios of the model shear wall structure in the X and Y directions were 1/568 and 1/687, which were less than the requirement of the MCE displacement angle limit of 1/200. The maximum story drift ratios of the steel truss structure were 1/358 and 1/185 in the X and Y directions, which were less than the requirement of the MCE displacement angle limit of 1/100 (Table 10). Although the structure had local damage, it still met the seismic performance target under the MCE with a large safety reserve.

**Table 10:** Model maximum story drift ratio.

Layer	SLE		MCE	
	X	Y	X	Y
1–7	1/2183	1/3532	1/1019	1/1152
8–16	1/2716	1/1684	1/696	1/681
17–23	1/2534	1/1665	1/739	1/637
24–25	1/1258	1/1341	1/468	1/587
26	1/2135	1/3587	1/1245	1/1143
27–30	1/821	1/455	1/258	1/185

### BRBs’ yield state

Due to the small yield displacement of BRBs, the yield strain was measured in the experiment to determine the state of BRBs under different levels of earthquake action. Table 11 shows the determination results of the BRB yield state at typical locations. According to Table 11, the BRBs did not yield under the SLE action; under the DBE action, some BRBs yielded; and under the MCE action, all BRBs yielded, proving that BRBs could meet the requirements of seismic design.

**Table 11:** BRB yield state.

Phase	BRB number	Strain	Yield strain	Yield or not
SLE	1B	75	164	No
	2B	93	166	No
	3B	105	246	No
	4B	61	164	No
	5B	51	166	No
	6B	76	246	No
	7B	63	205	No
	8B	104	164	No
DBE	1B	138	164	No
	2B	189	166	Yes
	3B	221	246	No
	4B	138	164	No
	5B	292	166	Yes
	6B	237	246	No
	7B	281	205	Yes
	8B	155	164	No
MCE	1B	386	164	Yes
	2B	459	166	Yes
	3B	434	246	Yes
	4B	356	164	Yes
	5B	473	166	Yes
	6B	610	246	Yes
	7B	583	205	Yes
	8B	466	164	Yes

## Conclusions

In this study, a 1/40 scale model of a large-span high-rise cliff hotel in a mountainous area was built and shaking table tests were conducted. In the tests, a steel frame with viscous dampers was designed to simulate the wave passage effect and slope amplification effect. Based on the test results, the damage, dynamic characteristics, and various dynamic responses of the structure were analyzed. The following conclusions could provide a reference for further research and practical engineering applications.

1. Three seismic waves were delayed by about 0.4 s and amplified by about 1.6 times after passing through the steel frame with viscous dampers. The goals of time-delay and amplification effects of input seismic waves were achieved in the test, showing that using a steel frame with viscous dampers to simulate the wave passage effect and slope amplification effect is feasible and practical.
2. Under the SLE action, the natural frequency of the scale model remained almost constant, with a maximum decrease of 0.6%, and the model was in an elastic state. The maximum story drift ratios in the X and Y directions of the shear wall structure were 1/1258 and 1/1341. The maximum story drift ratios in the X and Y directions of the steel truss structure were 1/821 and 1/455, which were less than the displacement angle limit values in the seismic performance target.
3. Under the DBE action, the natural frequency of the model significantly decreased, with a maximum decrease of 11.28%. The model began to fail, manifested explicitly as cracks appearing at the ends of some connecting beams and corners of openings.
4. Under the MCE action, the first three natural frequencies of the model decreased by more than 10%, indicating significant degradation of the lateral stiffness of the model. However, the previous damage further deepened, with cracks appearing in the shear wall and some components of the steel truss structure yielding, without severe damage to the overall structure. Moreover, the maximum story drift ratios in the X and Y directions of the shear wall structure were 1/568 and 1/687. The maximum story drift ratios in the X and Y directions of the steel truss structure were 1/358 and 1/185, which were less than the displacement angle limit values in the seismic performance target.
5. The BRBs in the model did not yield under the SLE, partially yielded under the DBE, and all yielded under the MCE. This shows that the BRBs played an important role in energy dissipation and seismic reduction under different seismic actions, meeting the expected seismic design goals.
6. Based on the performance of the model in the shaking table test, the prototype structure had good seismic performance, and met the requirements of seismic performance targets under different seismic intensities. Furthermore, adjusting the stiffness or mass distribution of the structure to increase the difference between the frequency of the protruding part and the frequency of the overall structure can reduce the influence of the whipping effect.
7. In this study, the seismic performance of a uniquely shaped large-span high-rise building was analyzed using a shaking table test, filling the research gap for this type of building. A designed steel frame was adopted to simulate the wave passage effect and slope amplification effect in the test, which provides new ideas and methods for similar shaking table tests. The conclusions of the tests in respect of the seismic performance of a large-span high-rise building can provide a basis for the design of similar buildings in the future. However, limited by the performance of the shaking table device, this test could not simulate vertical seismic action and the obtained test results were limited.

In future investigations, a finite element model can be established for the analysis of the prototype structure, and the numerical simulation results can be compared with the shaking table test results. This will enable better study of the seismic performance of the prototype structure. In addition, attempts can be made to adjust the viscous dampers in the steel frame to simulate the effects of amplifications and time-delays of different seismic waves on the seismic performance of buildings. A shaking table with better performance can be used to simulate vertical seismic action to make test results more accurate.

### Supplementary Information

Supplementary Information.

## Data Availability

The datasets used and/or analysed during the current study available from the corresponding author on reasonable request. All data generated or analysed during this study are included in this published article [and its supplementary information files].
